# Rapidly progressive Guillain–Barré syndrome following amitriptyline overdose and severe *Klebsiella pneumoniae* infection: A case report and literature review

**DOI:** 10.3389/fmed.2022.991182

**Published:** 2022-10-04

**Authors:** Boyu Zhang, Liwei Duan, Linhao Ma, Qingqing Cai, Hao Wu, Liang Chang, Wenfang Li, Zhaofen Lin

**Affiliations:** ^1^Department of Emergency and Critical Care Medicine, Changzheng Hospital, Naval Medical University, Shanghai, China; ^2^Department of Emergency Medicine, Shanghai Fourth People's Hospital Affiliated to Tongji University School of Medicine, Shanghai, China; ^3^Genoxor Medical & Science Technology Inc., Shanghai, China

**Keywords:** Guillain–Barré syndrome (GBS), *Klebsiella pneumoniae* infection, amitriptyline overdose, bilateral weakness, case report

## Abstract

Guillain–Barré syndrome (GBS) is a potentially life-threatening post-infectious autoimmune disease characterized by rapidly progressive symmetrical weakness of the extremities. Herein, we report a case of GBS associated with drug poisoning complicated by *Klebsiella pneumoniae* infection. A 38-year-old woman was admitted to the intensive care unit after taking an overdose of amitriptyline and was later diagnosed with coma, *Klebsiella pneumoniae* infection, and septic shock. Thirteen days after admission, she was diagnosed with GBS based on acute muscle pain, flaccid paralysis, hyporeflexia, reduced amplitude of compound muscle action potential, and albuminocytologic dissociation in the cerebrospinal fluid. GBS rarely occurs after a drug overdose and septic shock, and this is the first report of a rapidly progressive GBS following amitriptyline overdose and severe *Klebsiella pneumoniae* infection.

## Introduction

Guillain–Barré syndrome (GBS) is an acute, generalized polyradiculoneuropathy that can cause rapidly progressive flaccid weakness (1). Although its pathogenesis is not fully understood, most experts believe that it might be due to the recognition of antigens in the body by the immune system, which causes the autoimmune cells and antibodies to attack the peripheral nerves and cause peripheral nerve demyelination (2). Herein, we present a case of amitriptyline overdose complicated by *Klebsiella pneumoniae* infection-induced pneumonia and secondary GBS.

## Case description

**Figure 1:**
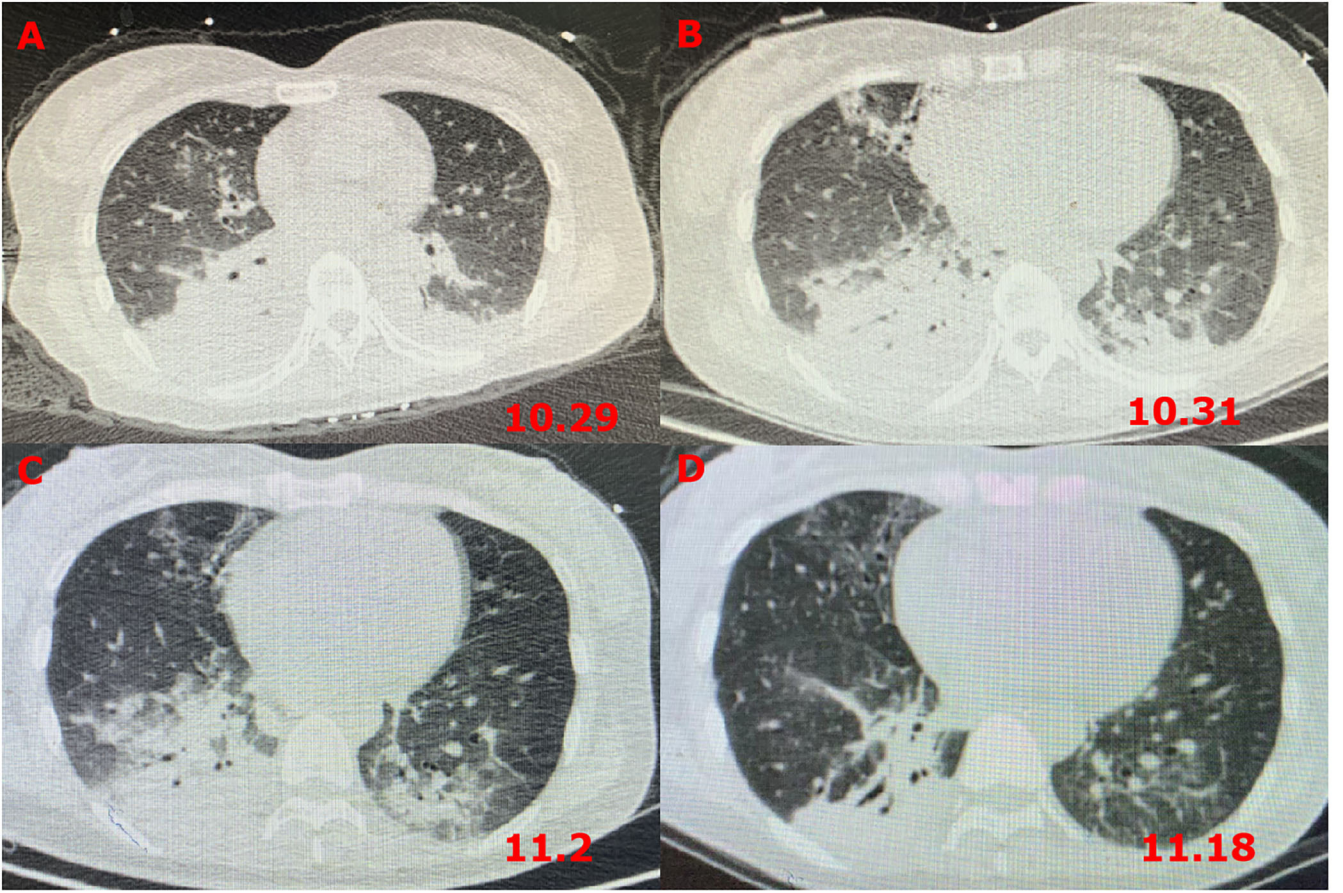
Chest CT images revealing pneumonia. **(A)** CT scans on the first day after admission; **(B)** CT scans at 72 h after admission; **(C)** CT scans on the fourth day after transferring out of the ICU; and **(D)** CT scans after GBS diagnosis and treatment for 5 days.

A 38-year-old woman, known to have had depression for over half a year, presented to the emergency department 6 h after taking an overdose of amitriptyline (about 100 tablets). On examination, she had a heart rate of 134 beats/min, a blood pressure of 110/66 mmHg, a respiratory rate of 21 breaths/min, a body temperature of 36.2°C, an oxygen saturation level of 98%, a Glasgow Coma Scale score of five, and a sequential organ failure assessment score of nine. Chest computed tomography (CT) (Figure 1) revealed inflammation in both the lungs, especially in the right lung, which was considered to be due to aspiration pneumonia, local bronchiectasis in the middle lobe of the right lung, and interstitial pulmonary edema in the upper lobe of both lungs. In addition, we were informed that the patient was allergic to penicillin and had no other significant medical or family genetic history.

On admission, the patient was immediately scheduled for emergency treatment. After gastric lavage, endotracheal intubation, and blood pressure control with vasoactive drugs, the patient was admitted to the intensive care unit (ICU). The remaining drugs were removed by hemoperfusion combined with hemofiltration adsorption. Meanwhile, moxifloxacin and biapenem were used to treat the infection and for organ support. After 2 days, the concentration of amitriptyline in the patient's blood decreased significantly (Table 1).

**Table 1:** Concentrations of amitriptyline in the patient's blood.

	**10.29 (Before adsorbent)**	**10.29 (After adsorbent)**	**10.31**	**11.1**
Amitriptyline	1.4 μg/ml	1.2 μg/ml	0.6 μg/ml	0.5 μg/ml
Olanzapine	14 ng/ml	15 ng/ml	5 ng/ml	4 ng/ml

**Figure 2:**
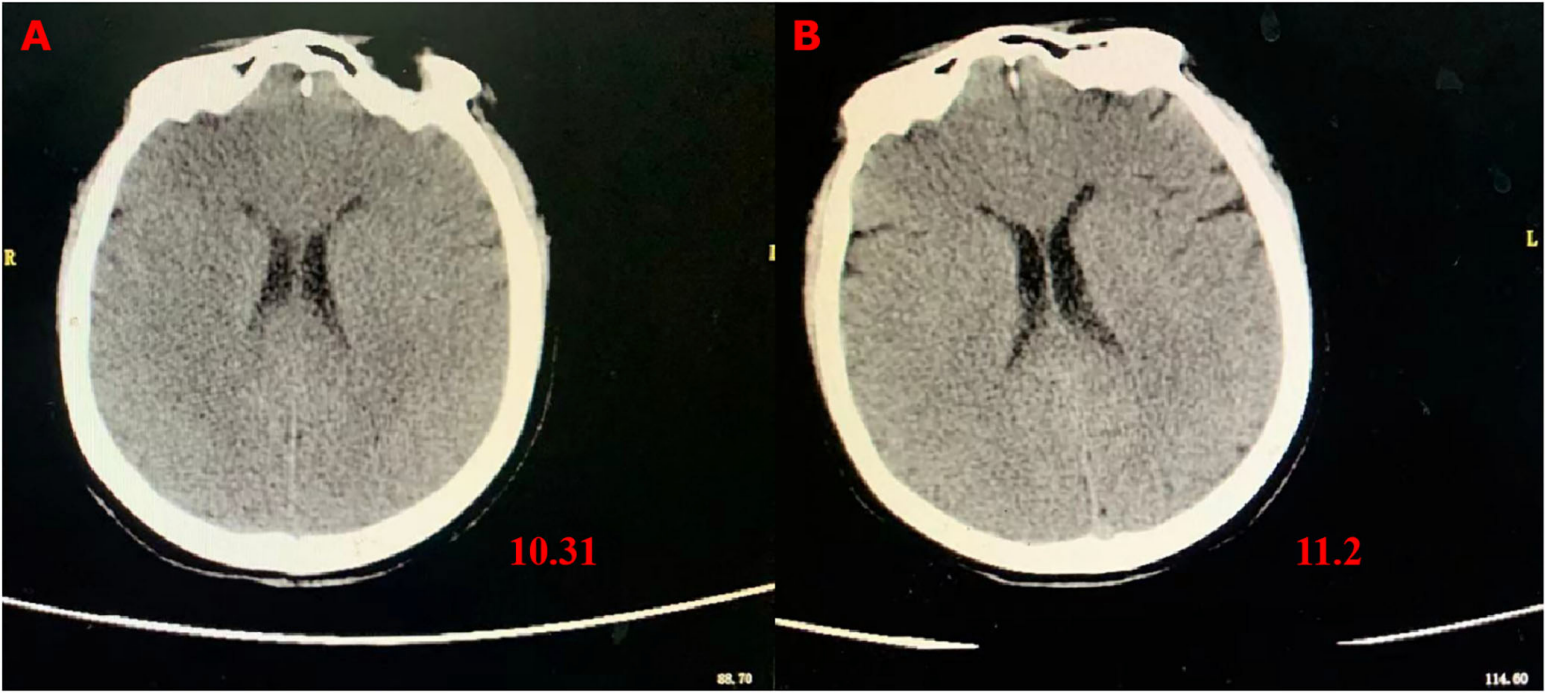
Skull CT images showing brain edema. **(A)** CT scans at 72 h after admission; **(B)** CT scans on the fourth day after transferring out of the ICU.

**Figure 3:**
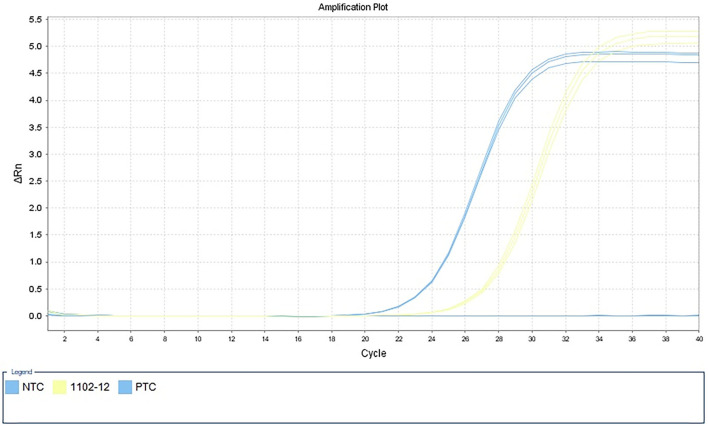
Detection of *Klebsiella pneumoniae* by qPCR in alveolar lavage fluid samples. NTC, negative control; 1102–12, alveolar lavage fluid sample; PTC, positive control.

However, 72 h after admission, her body temperature rose to 39.8°C, while her blood pressure and oxygen saturation continued to decline. The invasive hemodynamic assessment was performed by measuring pulse index continuous cardiac output, and the patient was diagnosed with septic shock in addition to amitriptyline overdose. Chest CT (Figure 1) showed progression of pneumonia, and skull CT (Figure 2) showed brain edema due to hypoperfusion. Metagenomic next-generation sequencing (mNGS) of the bronchoscope lavage fluid revealed the abundance of *Klebsiella pneumoniae* sequence, and the pathogen *Klebsiella pneumoniae* was confirmed by quantitative polymerase chain reaction (qPCR) (Figure 3). Imipenem and tigecycline were used as anti-infective agents, considering the possibility of septic shock caused by aspiration pneumonia. At the same time, other symptomatic treatments were administered, such as acid suppression, liver protection, phlegm resolution, white protein supplementation, diuresis, rehydration, and potassium supplementation through micro-pumps, fluid infusion, and nutritional support. On the second day of treatment, the patient showed substantial clinical improvement. The blood gas analysis results were satisfactory after endotracheal intubation was removed, and the patient was transferred out of the ICU after 5 days.

Unfortunately, the patient gradually developed symmetrical limb weakness, which continued to worsen and was primarily diagnosed as ICU-acquired weakness. The patient could neither move her limbs nor eat independently for 4 days despite being transferred out of the ICU. Physical examination showed that the limb muscle strength level was within the range of grade 0–1, muscle tone was decreased, expectoration could not be produced, and sputum was abundant and viscous. Ceftazidime–avibactam sodium was administered as an anti-infective treatment (2.5 g q8h). Cerebrospinal fluid was collected through a lumbar puncture on the fifth day for laboratory examination and diagnosis. The results confirmed protein cell separation; cerebrospinal fluid protein levels were 1,104 mg/L, and cerebrospinal fluid leukocytes were 3 × 10^6^/L. The nerve conduction study test showed severely slowed conduction velocities with disappeared compound motor action potential and absent H-reflexes and F-waves, thus suggesting demyelination of nerves. The antibodies in cerebrospinal fluid against autoimmune peripheral neuropathy and myasthenia gravis were absent. Therefore, these symptoms could not be explained by ICU-acquired weakness and sepsis secondary to multiple myopathies, which have normal findings on the nerve conduction study tests. Based on the results of these tests and consultation with neurology experts, the patient was diagnosed with secondary GBS.

**Figure 4:**
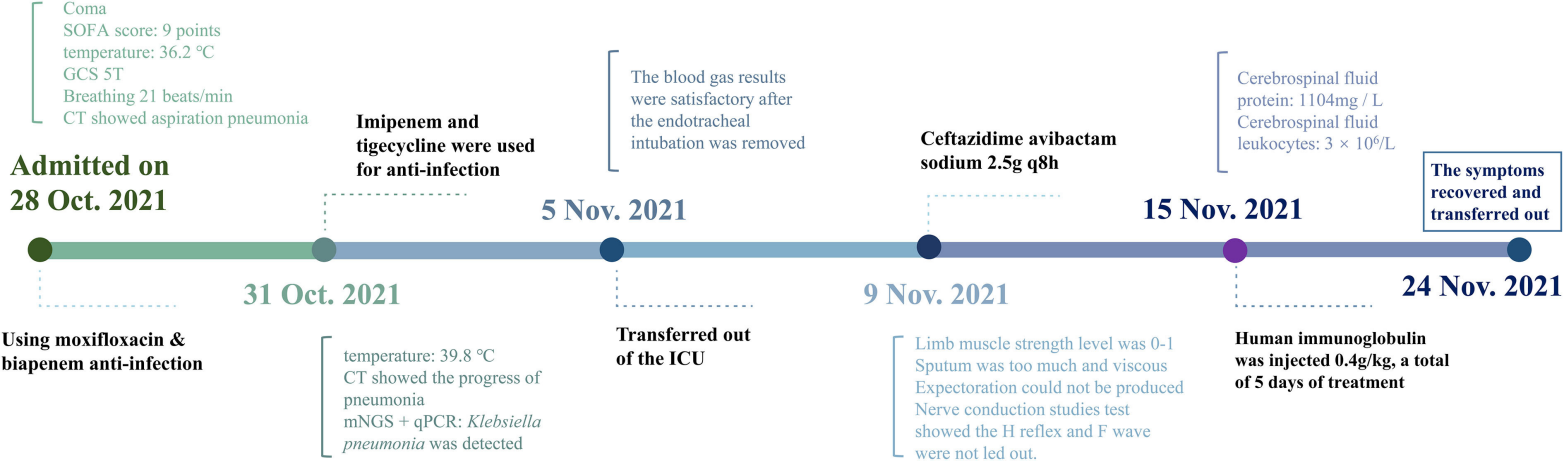
Diagnosis and treatment timeline of the case.

Afterward, human immunoglobulin was administered at 0.4 g/kg body weight/day (daily dose, 20 g; calculated according to her body weight) for 5 days, with close monitoring of the patient's respiratory status and supplementation by symptomatic support treatment. After 5 days, the patient's mental state improved. Her voice was louder, and she could move her fingers and toes slightly. Furthermore, the infection index and chest CT (Figure 1) showed significant clinical improvement. She was subsequently transferred to a rehabilitation hospital for maintenance therapy 2 weeks later. Four weeks after she was discharged, the muscle strength of the distal extremities was within the range of grade 1–2, and the symptoms gradually resolved. The timeline of the patient's clinical course described above is presented in Figure 4.

## Discussion

### Pathogen infection might be a crucial essential factor causing GBS

Two-thirds of patients with GBS have prior respiratory or gastrointestinal symptoms. Usually, an abnormal autoimmune response is induced in the peripheral nerve and the spinal cord root due to infection or other immune stimulation, which leads to the development of GBS (1, 2). Infection with severe acute respiratory syndrome coronavirus 2 (SARS-CoV-2), *Campylobacter jejuni*, cytomegalovirus, Epstein–Barr virus, *Haemophilus influenzae, Mycoplasma pneumoniae*, herpes simplex virus, and *Borrelia burgdorferi* (Lyme disease) are reportedly associated with the pathogenesis of GBS (3). GBS has also been reported to occur following SARS-CoV-2 vaccination (4). In this case, we believe that the coma after drug poisoning caused aspiration pneumonia by *Klebsiella pneumoniae*. According to the findings of this case of severe respiratory tract infection caused by *Klebsiella pneumoniae*, the diagnosis of GBS should be considered if the patient shows clinical symptoms such as symmetrical limb weakness.

### Clinical classification and diagnostic criteria for GBS

GBS is characterized by rapid progression and symmetrical limb weakness with hyporeflexia or the disappearance of reflexes but has not been reported to be caused by inhalation infection after drug poisoning. Its clinical causes, sensory symptoms, muscle weakness, ataxia, pain, and autonomic nerve dysfunction are highly variable (5). According to its clinical signs and electrophysiological diagnostic characteristics, GBS can be divided into four subtypes: acute motor axonal neuropathy, acute motor and sensory axonal neuropathy (AMSAN) with both motor and sensory involvement, acute inflammatory demyelinating polyneuropathy characterized by demyelinating lesions and sensory disorders, and Miller Fisher syndrome dominated by facial paralysis (2, 3). BS diagnosis depends mainly on clinical symptoms, followed by cerebrospinal fluid evaluation and electromyography. Different subtypes have different clinical manifestations, electrophysiology, and histopathology. For typical GBS, rapid progressive bilateral limb weakness is the main symptom. In other subtypes, patients show cranial nerve function defects, especially bilateral facial muscle weakness, dysphagia, or extraocular muscle dyskinesia. Some patients experience respiratory failure or severe autonomic nerve dysfunction. A slight increase in leukocyte count may be found in cerebrospinal fluid analysis. However, due to the dissociation of albumin cells, the protein count in the cerebrospinal fluid remains very high (2, 6). In this study, despite actively controlling the condition, symmetrical weakness of the limbs gradually increased, resulting in the patient being unable to move her limbs or eat independently. The limb muscle strength level of the distal extremities was within the range of grade 0–1, the muscle tone was decreased, and she could not expectorate, which was consistent with the symptoms of AMSAN (2, 7). Her diagnosis of GBS is a diagnosis of exclusion, as the primary attempt of our in-depth workup was to exclude all other likely diagnoses that would explain her clinical presentation. Demyelination was detected upon nerve conduction study, and this could be used to distinguish GBS from ICU-acquired weakness. The antibodies in cerebrospinal fluid against autoimmune peripheral neuropathy and myasthenia gravis were absent, so multiple myopathies were excluded from the diagnosis. After the GBS diagnosis was confirmed, and intravenous immunoglobulin was administered, the patient showed noticeable improvement (8).

### Immunomodulatory therapy is the primary clinical treatment

Multidisciplinary cooperation and immunotherapy are often used to treat GBS, with plasma exchange (PE) therapy and intravenous immunoglobulin being the effective methods (7). PE therapy within the first 4 weeks of onset has been reported as a proven effective treatment method for GBS. As intravenous immunoglobulin is also easier to administer and is more widely available than plasma exchange, it is usually the treatment of choice (8–10). PE acts by reducing or removing specific antibody components in the blood, thereby reducing immune cross-reactions. Usually, in patients with GBS, PE therapy is performed three to five times every other day for 7–14 days, and the PE rate is 120–200 ml/kg (40–50 ml/kg/day) (11). Similarly, intravenous injection of high-dose immunoglobulin (in the present case, the dose administered was 0.4 g/kg body weight/day for 5 days) can reduce the concentration of specific antibodies in the patient's blood by increasing the amount of immunoglobulin and reducing the antibody response to pathogenic antigens (12). Its curative effect was reported to be similar to that of PE therapy (13, 14). Intravenous injection of gamma globulin usually begins within 2 weeks of GBS onset. Early initiation of intravenous immunoglobulin or PE is beneficial and crucial, especially in patients with rapidly progressive weakness (11, 15). Previous studies showed that following the progression stage of the disease is the plateau stage, which usually lasts for 2 days−6 months (average, 7 days), after which the patient starts to recover (2, 3, 7). In the present case, because of the timely diagnosis, a 5 day human immunoglobulin dose of 0.4 g/kg body weight/day was administered. We significantly controlled the development of the disease and, thus, had adequate time for follow-up rehabilitation.

## Conclusion

We reported a case of amitriptyline overdose complicated by severe pneumonia caused by *Klebsiella pneumoniae* infection and rapidly progressive GBS. It is necessary to distinguish cases of the unexplained decline of limb muscle strength or abnormal sensations from easily confused conditions, such as ICU-acquired weakness and multiple myopathies, and be alert to possible GBS. Careful medical history, examination of systemic nervous function, cerebrospinal fluid examination, detection of pathogenic microbes by mNGS, magnetic resonance imaging of the head and the neck, electromyography, and autoantibody spectrum of autoimmune peripheral neuropathy should be considered for precise diagnosis. In addition, predictive biomarkers need to be developed to achieve better results for faster identification and to guide diagnosis and treatment.

## Data availability statement

The original contributions presented in the study are included in the article/supplementary material, further inquiries can be directed to the corresponding author.

## Ethics statement

Written informed consent was obtained from the patient for the publication of any potentially identifiable images or data included in this article.

## Author contributions

BZ collected clinical data and wrote the manuscript. QC guided mNGS, qPCR experiments, and data analysis. LD, HW, LC, WL, and ZL participated in the clinical care of the patient and collected a large body of literature to support this diagnosis. WL and ZL assisted in interpreting the results from a clinical perspective and provided suggestions for later revision of the article. LM designed the study and critically revised the manuscript for intellectual content. All authors have read and approved the final manuscript.

## Conflict of interest

Author QC is employed by Genoxor Medical & Science Technology Inc., Shanghai, China.

The remaining authors declare that the research was conducted in the absence of any commercial or financial relationships that could be construed as a potential conflict of interest.

## Publisher's note

All claims expressed in this article are solely those of the authors and do not necessarily represent those of their affiliated organizations, or those of the publisher, the editors and the reviewers. Any product that may be evaluated in this article, or claim that may be made by its manufacturer, is not guaranteed or endorsed by the publisher.
